# Checkpoint Kinase 2 (CHEK2) Gene Mutation in a Patient With Breast and Prostate Cancer: A Unique Presentation of a Rare Disease

**DOI:** 10.7759/cureus.49710

**Published:** 2023-11-30

**Authors:** Samar N Ekram, Nasser Al Shanbari, Bassam M Bin Laswad, Abdulrahman Alharthi, Waseem Tayeb, Abdulbari Bahha

**Affiliations:** 1 Department of Medical Genetics, College of Medicine, Umm Al-Qura University, Makkah, SAU; 2 Department of Medicine and Surgery, College of Medicine, Umm Al-Qura University, Makkah, SAU; 3 Department of Surgery, Division of Urology, King Abdullah Medical City, Makkah, SAU

**Keywords:** cancer genetics, prostate cancer, male breast cancer, chek2 gene, gene mutation

## Abstract

Breast cancer is one of the rarest malignancies in males, with a low incidence rate compared to all breast cancers. Gene mutation plays a significant role in the pathologic process of cancer. Mutations in breast cancer gene 1 (*BRCA1*) and breast cancer gene 2 (*BRCA2*) have been associated with male breast cancer (MBC), as well as prostate cancer (PCa). Despite the etiopathogenetic similarity, combined MBC and PCa is a rare entity.

This report presents the case of a 57-year-old male with a history of breast cancer who underwent modified radical mastectomy (MRM) with lymph node dissection followed by adjuvant chemoradiotherapy four years ago. The patient presented with recurrent episodes of voiding dysfunction for three months, followed by urine retention. His family history was positive for breast and lung cancer. High prostate-specific antigen (PSA) and Prostate Imaging-Reporting and Data System 5 (PI-RADS5) necessitate transrectal ultrasound-guided biopsy, which confirmed the diagnosis of PCa. Molecular genetics testing and next-generation sequencing (NGS) analysis identified heterozygous variant c.636T>G, p.(Tyr212*) in the checkpoint kinase 2 (*CHEK2*) gene. The patient is planned for neoadjuvant luteinizing hormone-releasing hormone (LHRH) for 3-6 months, to be followed by transurethral tunneling of the prostate (TUTP) with adjuvant LHRH. The allele frequency of this patient mutation was documented for the first time among the general population, and it has not been described in the literature. This unique and rare case was presented with clinical, morphological, and immunohistochemical features together with a review of the current literature.

## Introduction

Breast carcinoma is rare to be seen in males, where it represents only 1% of all breast cancers [1]. Male breast cancer (MBC) has multiple risk factors, including old age, high estrogen levels, and diagnoses of breast, prostate, or ovarian cancer in a first-degree relative [2]. However, in 4%-40% of the cases, pathogenic mutations in cancer-predisposing genes are a possible etiology for MBC [3].

On the other hand, prostate cancer (PCa) is the most common cancer in males, accounting for about 25% of all male cancers and the second cause of cancer-related mortality in males [4]. Both MBC and PCa have been associated with gene mutations such as breast cancer gene 1 (*BRCA1*) and breast cancer gene 2 (*BRCA2*) germline mutations [5]. Although the etiopathogenetic characteristics of MBC and PCa are quite similar, their combination is unique [1]. Furthermore, there are sparse data about the association between both MBC and PCa and their genetic features. Therefore, this report will present a unique and rare case of metachronous MBC and PCa, with a review of the current literature.

## Case presentation

**Figure 1:**
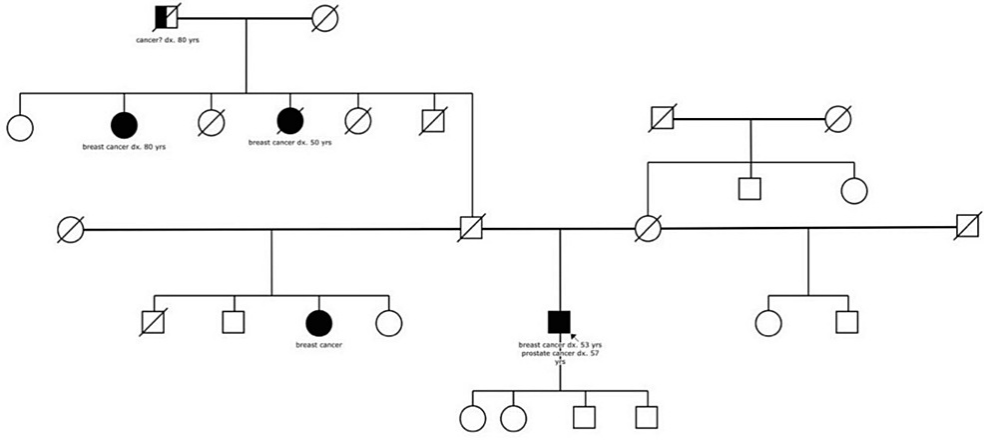
Family pedigree: proband is marked with an arrow

A 57-year-old male was presented to a tertiary care hospital with bothersome voiding dysfunction and recurrent episodes of urine retention for a three-month duration. The patient is a known case of type 2 diabetes mellitus, hypertension, and dyslipidemia, which are managed by metformin, valsartan, and atorvastatin, respectively. He has a previous history of left breast cancer, which was diagnosed at the age of 53 years old and managed by modified radical mastectomy (MRM), followed by combined adjuvant chemotherapy and radiotherapy, and he is currently on adjuvant tamoxifen. The patient’s paternal aunt was diagnosed with breast cancer at the age of 80 years, and another aunt died of breast cancer at the age of 50 years. The patient’s paternal grandfather died from lung cancer at the age of 80 years, and one of his half-sisters (paternal side) was diagnosed with breast cancer (Figure 1). He is married and works as a teacher and has two healthy sons and two daughters. The patient did not report any history of allergy to food or drugs and never smoked tobacco.

**Figure 2:**
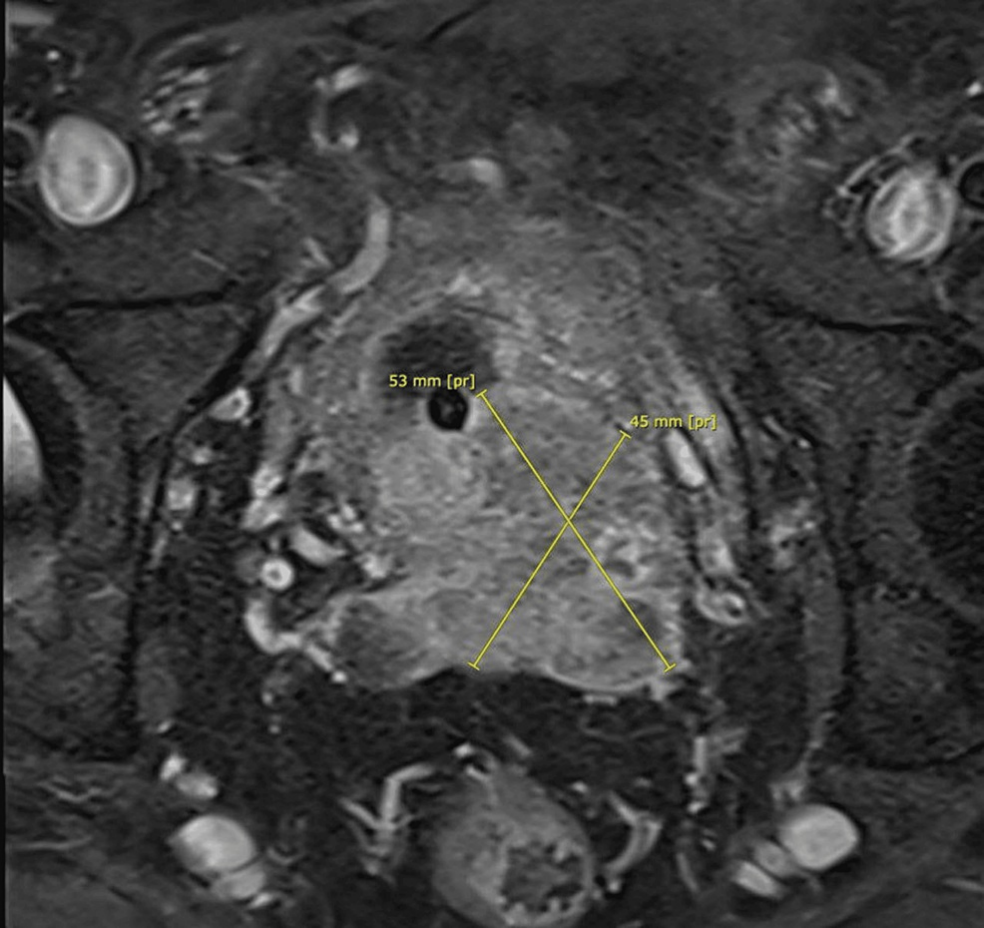
MRI of the prostate with contrast showing PI-RADS5 prostate lesion Diffusely enlarged prostate measuring 5.5 × 4.5 × 6.5 cm, with a volume of 80 g, demonstrates heterogeneous ill-defined lobulated abnormal signal intensity lesion about 5 cm in diameter of moderate T2 homogeneous hypointensity involving mainly the transition zone, extending to the bladder base, seminal vesicles, extra-prostatic left posterolateral neurovascular bundle, and abutting left levator ani, mainly involving the left side of the prostatic base MRI, magnetic resonance imaging; PI-RADS5, Prostate Imaging-Reporting and Data System 5

**Figure 3:**
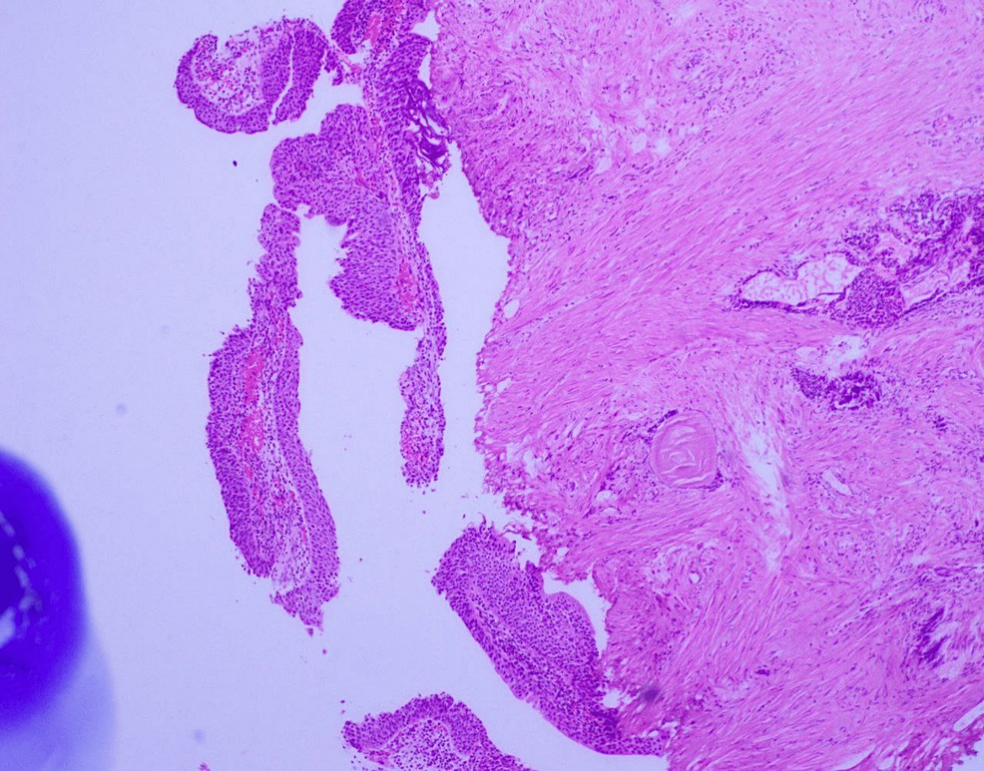
Transrectal ultrasound-guided biopsy of the prostate showing bilateral acinar adenocarcinoma

The patient was diagnosed with PCa by a high prostate-specific antigen (PSA) of 162 ng/mL and magnetic resonance imaging (MRI) of the prostate, which showed Prostate Imaging-Reporting and Data System 5 (PI-RADS5), prostate lesion of 5 cm, locally infiltrating the seminal vesicles, urinary bladder base, extra-prostatic left posterolateral neurovascular bundle, and abutting left levator ani with two suspicious meso-rectal lymph nodes (Figure 2). Transrectal ultrasound-guided biopsy demonstrated bilateral acinar adenocarcinoma, Gleason score of 9 (4 + 5), grade group 5, and tumor that involved 90% of the right lobe and 50% of the left prostate lobe (Figure 3).

The patient has completed a course of luteinizing hormone-releasing hormone (LHRH) agonists and enzalutamide therapy for six months. Afterward, the follow-up MRI showed interval regression in the prostatic mass size with no pelvic lymphadenopathy. The plan was to repeat neoadjuvant LHRH for 3-6 months, to be followed by transurethral tunneling of the prostate (TUTP) with adjuvant LHRH.

Furthermore, the patient underwent molecular genetic testing of breast cancer-predisposing genes, including ataxia-telangiectasia mutated (*ATM*), *BRCA1*-associated RING domain 1 (*BARD1*), *BRCA1*, *BRCA2*, *BRCA1* interacting protein C-terminal helicase 1 (*BRIP1*), cadherin 1​​​​​​​ (*CDH1*), checkpoint kinase 2 (*CHEK2*), meiotic recombination 11 homolog A (*MRE11A*), nibrin (*NBN*), partner and localizer of *BRCA2*​​​​​​​ (*PALB2*), RAD51 paralog D (*RAD51D*), serine/threonine kinase 11​​​​​​​ (*STK11*),* *and tumor protein p53​​​​​​​* *(*TP53*). Next-generation sequencing (NGS) analysis identified the heterozygous variant c.636T>G, p.(Tyr212*) in the *CHEK2* gene. This pathogenic variant increases the risk of breast cancer. Molecular genetic testing and genetic counseling were offered to the patient and his relatives through our geneticist.

## Discussion

This is a case of a 57-year-old male who was interestingly diagnosed with metachronous MBC and PCa. In addition, he had a significant family history of breast and lung cancer. Molecular genetic testing confirmed the association of a heterozygous mutation c.636T>G, p.(Tyr212*) in the *CHEK2* gene, which results in a premature stop codon and subsequent mRNA degradation (nonsense-mediated decay) leading to a truncation (shortening) of the protein. The allele frequency of this patient mutation was documented for the first time among the general population (Genome Aggregation Database {gnomAD} v2.1.1 controls), and it has not been described in the literature (Human Gene Mutation Database {HGMD} 2020.3). The result of the genetic testing exhibits the possibility that this heterozygous pathogenic variant might be the cause of the patient’s tumors. Furthermore, the mode of inheritance of his mutation is autosomal dominant (AD), which shows a 50% probability that the patient would transmit the *CHEK2* variant c.636T>G, p.(Tyr212*) to his offspring. Therefore, genetic counseling was offered to the patient and his family [6].

Breast cancer can be caused by the interaction of multiple factors including environmental and genetic factors [7]. A positive family history of breast cancer increases the individual’s risk for developing breast cancer threefold [8]. Furthermore, hereditary factors have been reported as the cause of 5%-10% of all breast cancer cases [8]. However, PCa represents one of the highest cancers in incidence and mortality rates worldwide, including 10%-20% of cases, which are caused by genetic factors [9]. There are many genes that were linked to PCa, which have also been related to common hereditary cancer syndromes, including breast and ovarian cancer (*BRCA1*, *BRCA2*, *ATM*, *CHEK2*, and *PALB2*) and Lynch syndrome (*MLH1*, *MSH2*, *MSH6*, and *PMS2*) [9].

*BRCA1* and *BRCA2* have also been linked with an increased risk for MBC, accounting for 1%-2% and 5%-10%, respectively [10]. In addition to *BRCA1/2*, other genes have been associated with a fourfold increase in breast cancer risk including *TP53*, *PALB2*, *ATM*, and *CHEK2* [11]. The mutation of the *CHEK2* gene has been associated with numerous types of cancers, including breast cancer and PCa [7]. Moreover, the risk of breast cancer increases twofold with the *CHEK2* 1100delc mutation [12]. Also, the *CHEK2* 1100delc mutation has been found in four of 68 patients who were diagnosed with MBC in the Finnish population [12]. Additionally, the *CHEK2* 1100delC mutation or *CHEK2* I157T missense is associated with an increased risk of prostate cancer, and the mutation of *CHEK2* is certainly related to the development of prostate cancer [13].

The risk of breast cancer among *CHEK2* gene mutation carriers differs according to family history of cancer [14]. Although breast cancer has a low incidence rate in males of 1:1000, it may be elevated in patients with a genetic predisposition to breast cancer [15]. Moreover, it has been documented by a Polish study that the mutations of *CHEK2*, *BRCA1*, *BRCA2*, *PALB2*, and *NBN* were found among 13.3% of males diagnosed with breast cancer [16]. A recent study reported that the *CHEK2* gene was the second most frequent mutation detected among patients with metastatic PCa [17].

According to recent reports, *CHEK2* gene mutations have been associated with numerous types of cancer; in a 37-year-old female patient who was diagnosed with bilateral breast cancer, it was associated with compound heterozygous frameshift variants in *CHEK2 *gene: c.277del (p.Trp93Glyfs*17) and c.902del (p.Leu301Trpfs*3) [18]. Moreover, *CHEK2* 1100delC mutation has been found in a case of cholangiocarcinoma that was diagnosed in a 31-year-old female concurrently with c.1227_1228dupGG *MUTYH* gene mutation [19]. Additionally, the first association of *CHEK2* gene mutation with central nervous system tumors was reported recently in an oligodendroglioma patient [20]. *CHEK2* c.1100delC variant has been also reported in two sisters diagnosed with multiple endocrine tumors [21].

*CHEK2* gene is a tumor suppressor gene, which was discovered in 1998 and is located on the long arm of chromosome 22 at 22q12.1 and consists of 14 exons and 543 amino acids [22]. This gene encodes the CHK2 protein, a serine-threonine kinase that helps in maintaining the human genome through its critical role in DNA repair, cell cycle arrest at checkpoint, or apoptosis in response to DNA damage [23]. Thus, mutations to this gene have been linked as a cause of a wide variety of cancers [24]. Accordingly, it is strongly recommended to encourage those people at high risk to undergo screening program to prevent or detect early these cancers.

The rarity of the disease is limiting the studies, which assess the association between MBC, PCa, and *CHEK2* gene mutation [25]. Therefore, the current report contributes to the efforts of documenting and studying rare genetic mutations and its relation to male breast cancer and prostate cancer.

## Conclusions

Numerous gene mutations have been associated with hereditary cancers. The current report is a unique case of *CHEK2* gene mutation associated with metachronous breast and prostate cancer. This case report contributes to the efforts of studying and documenting hereditary cancers and their causative gene mutations. Moreover, this report emphasizes the importance of raising the awareness of the general population about cancer genetics and genetic testing for people who are at high risk of developing hereditary cancer to avoid unnecessary medical and surgical interventions and poor prognosis.
